# Periodically pulsed wet annealing approach for low-temperature processable amorphous InGaZnO thin film transistors with high electrical performance and ultrathin thickness

**DOI:** 10.1038/srep26287

**Published:** 2016-05-20

**Authors:** Ye Kyun Kim, Cheol Hyoun Ahn, Myeong Gu Yun, Sung Woon Cho, Won Jun Kang, Hyung Koun Cho

**Affiliations:** 1 School of Advanced Materials Science and Engineering, Sungkyunkwan University, 2066 Seobu-ro, Jangan-gu, Suwon, Gyeonggi-do, 16419, Republic of Korea

## Abstract

In this paper, a simple and controllable “*wet pulse annealing*” technique for the fabrication of flexible amorphous InGaZnO thin film transistors (*a*-IGZO TFTs) processed at low temperature (150 °C) by using scalable vacuum deposition is proposed. This method entailed the quick injection of water vapor for 0.1 s and purge treatment in dry ambient in one cycle; the supply content of water vapor was simply controlled by the number of pulse repetitions. The electrical transport characteristics revealed a remarkable performance of the *a*-IGZO TFTs prepared at the maximum process temperature of 150 °C (field-effect mobility of 13.3 cm^2^ V^−1^ s^−1^; I_on_/I_off_ ratio ≈ 10^8^; reduced I-V hysteresis), comparable to that of *a*-IGZO TFTs annealed at 350 °C in dry ambient. Upon analysis of the angle-resolved x-ray photoelectron spectroscopy, the good performance was attributed to the effective suppression of the formation of hydroxide and oxygen-related defects. Finally, by using the wet pulse annealing process, we fabricated, on a plastic substrate, an ultrathin flexible *a*-IGZO TFT with good electrical and bending performances.

The enhancement of the field-effect mobility and electrical stability in amorphous oxide semiconductors is an essential research topic that has recently received great attention in relation to next-generation flat panel displays^1^,^2^, including active-matrix liquid crystal displays and organic light-emitting diode displays^3^, as well as flexible electronic applications^4^,^5^. In particular, low-temperature processable amorphous InGaZnO (*a*-IGZO) thin films appear very promising, owing to their large area uniformity and the possibility to use flexible substrates, as well as their high field-effect mobility and low off-current level^6^,^7^,^8^.

Regarding flexible and imperceptible *a*-IGZO thin film transistor (TFT) devices, high TFT performance and high stability are very critical issues when a high thermal budget is involved^9^, as a low annealing temperature significantly degrades the TFT performances or prevents hard saturation^10^. The increase in the thermal annealing temperature results in internal modifications related to an improved local atomic rearrangement, which is possibly attributed to the changes in the oxidation states associated with metal-oxygen bonding, oxygen vacancies^11^, hydroxide formation, etc^12^. Typical approaches to obtain a stable metal oxide bonding and appropriate electrical conductivity involve the control of the Ar/O_2_ gas ratio during sputtering deposition^13^, thermal annealing in O_2_^14^, air, and N_2_ environments^15^, microwave^16^ or illumination treatments^17^, and high oxygen pressure^18^ or water vapor (H_2_O)^19^/ozone (O_3_)^20^ annealing. Among them, H_2_O, O_3_, and hydrogen peroxide have a stronger oxidizing power than O_2_, and thus they have been actively utilized as atmospheres for thermal annealing process^19^,^20^. In particular, it is reported that the use of H_2_O at high thermal annealing temperatures effectively reduced the presence of oxygen-related defects acting as subgap states within the band gap^21^,^22^.

Nevertheless, whether the use of H_2_O leads to positive or negative effects is still under debate. Some reports explained that annealing in a wet atmosphere led to a lower bias stress stability/negative bias temperature illumination stability or highly conductive channel^23^ due to unwanted hydrogen-related bonding. In addition, water exposure under high humidity conditions may lead to large hysteresis^24^ and variation in negative bias temperature illumination tests^25^, due to the action as a shallow acceptor-like trap. Generally, for high-temperature annealing above 300 °C, dry annealing in oxygen-rich or air ambient has been generally adopted as a reproducible optimum process to simultaneously obtain high performance and high stability^19^. On the other hand, flexible and imperceptible TFTs fabricated at low temperatures require an alternative post-treatment process. The O_2_ ambient is not suitable for low-temperature (below 200 °C) processed *a-*IGZO TFTs^26^, owing to the insufficient thermal energy. As a result, our strategy entailed the effective use of H_2_O, which has a strong oxidizing power, by retaining adequate wet ambient conditions. An appreciable increase in the oxidation rate for H_2_O in metal oxides was expected, probably due to the reaction of M–O with H_2_O to give –OH and the small sized H_2_O molecule. In this work, we proposed the “*wet pulse annealing*” technique for low-temperature processable *a-*IGZO TFTs as a valuable alternative to the conventional dry and wet annealing processes. This process consisted of an artificially periodic exposure to a wet ambient (H_2_O vapor) and a dry atmosphere (vacuum purge). The suggested annealing process could avoid excessive exposure due to a continuous wet injection, and the amount of H_2_O could be simply controlled by varying the injection parameters. The resultant *a-*IGZO TFTs fabricated at the maximum temperature of 150 °C exhibited quite good electrical properties and stability. Moreover, we successfully employed this approach to fabricate ultrathin *a-*IGZO TFTs on a flexible polymer with the thickness of a few microns.

## Results

**Figure 1:**
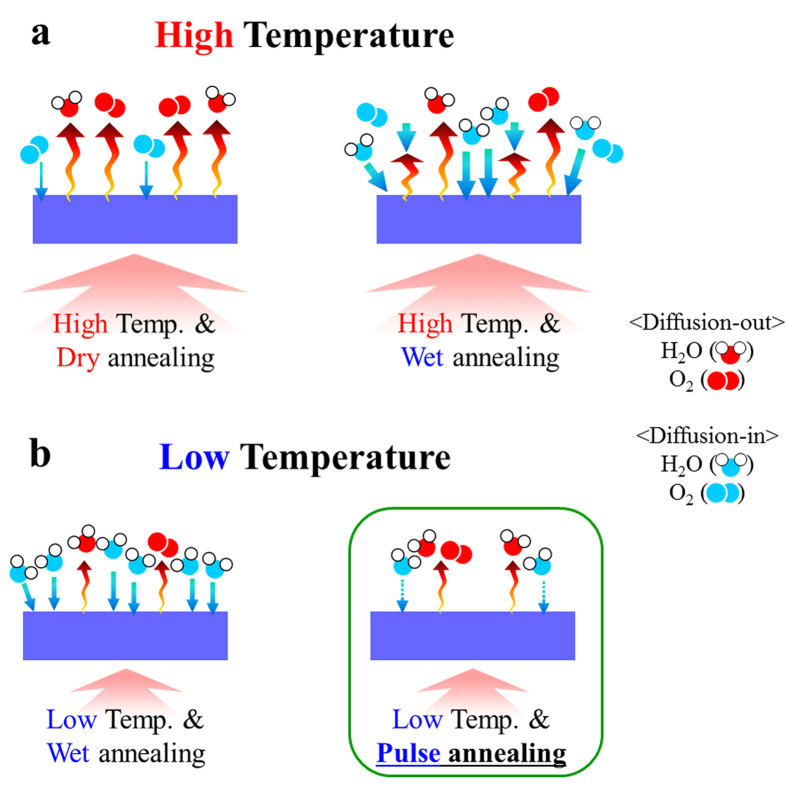
Schematic illustration of oxygen-related gas in/out diffusion mechanisms depending on thermal annealing temperature and gas ambient. (**a**) High temperature (>300 °C): dry and wet ambient. (**b**) Low temperature (<200 °C): wet and wet pulse ambient.

In a previous study, Hosono group reported that H_2_O molecules exhibited considerably higher surface reactivity in *a-*IGZO films than O_2_ molecules, and the surface reactivity was suppressed by wet annealing at 400 °C^26^. Typically, OH-related species, O_2_ molecules, Zn–O components, and H_2_O molecules can be desorbed or diffused depending on the annealing temperature, ambient, and time^19^. Notably, the wet environment suppressed the desorption of H_2_O, Zn, and O_2_ species, resulting in the variation of the electrical conductivity of the *a-*IGZO films. Consequently, the adoption of a wet ambient can be a very effective approach for the control of the desorption and absorption of some molecular species. However, until now, most studies have focused on the annealing temperature and time in wet ambient above 300 °C^19^. As shown in Fig. 1a, in the high-temperature region, the desorption of oxygen-related molecules such as O_2_ and H_2_O dominates over the diffusion-in process in the dry ambient; thus, wet annealing using H_2_O is an effective method to suppress the desorption and annihilate point defects, when H_2_O is continuously injected with constant supply. However, an excessive supply of H_2_O induces the formation of OH-related species in *a-*IGZO, causing a decrease in electrical conductivity and lower field-effect mobility in *a-*IGZO TFTs, owing to the well-known role of hydroxyl groups as trap sites for electrons^27^,^28^. In low-temperature annealing processes for flexible and imperceptible devices (Fig. 1b), the continuous supply of H_2_O may introduce an excess of hydroxyl bonding, due to the reduced desorption events; thus, adequate amounts of H_2_O should be provided to achieve an optimum trade-off between the effective supply of oxygen molecules via H_2_O for strong M-O bonding and the extent of unwanted hydroxyl bonding inducing low field-effect mobility during the low-temperature thermal annealing process, as shown in Fig. 1b 29. Our strategy for low-temperature processed *a-*IGZO TFTs with high performance and high stability using vacuum deposition involved the introduction of a wet pulse annealing process to artificially control the supply content of H_2_O during thermal annealing; the technique implied the use of periodic wet vapor inputs and dry purges.

**Figure 2:**
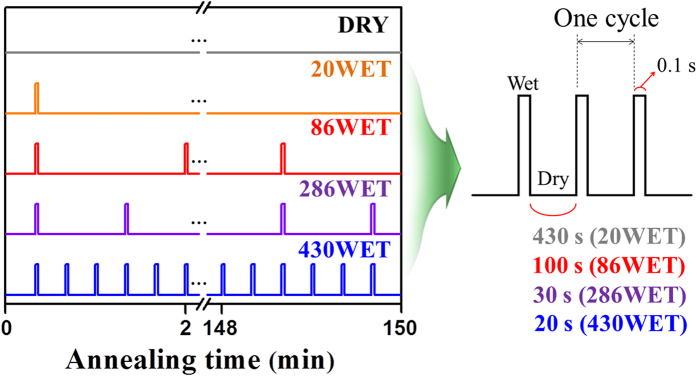
Design of pulsed wet annealing process consisting of periodic (0.1 s) water vapor injection and vacuum dry purge. Here, the water vapor injection numbers equal to 20, 86, 286, and 430 for 150 min.

The annealing temperature and time for the TFT fabrication were set to 150 °C and 150 min, respectively. As shown in Fig. 2, a quick injection of H_2_O for 0.1 s corresponded to the wet ambient; subsequently, the dry thermal treatment was conducted under vacuum with different purge times during the pulse annealing of one cycle. Among the various purge times, we representatively selected two cases with purge times of 430 s, 100 s, 30 s, and 20 s, which corresponded to injection numbers of 86 and 430 within 150 min; we named these samples “20WET”, “86WET”, “286WET”, and “430WET”, respectively. For comparison with the wet pulse annealed *a-*IGZO TFTs, a typical dry-annealed sample was also prepared (named “DRY”).

**Figure 3:**
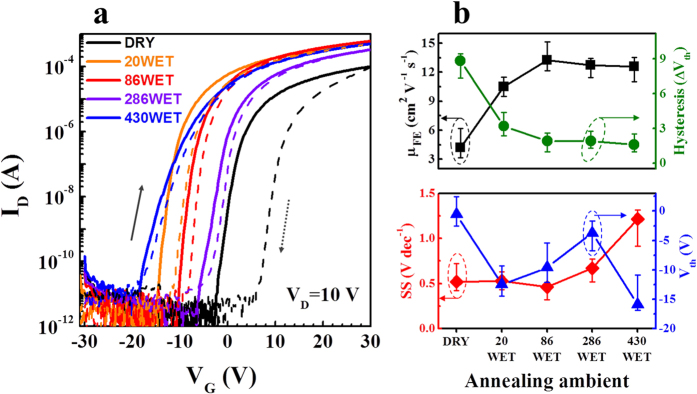
(**a**) Transfer characteristics of DRY (dry-annealed sample), 20WET (injection number: 20), 86WET (injection number: 86), 286WET (injection number: 286), and 430WET (injection number: 430) thin film transistors (TFTs) at V_D_ = 10 V. (**b**) Summary of the performance parameters of the DRY and WET annealed amorphous IGZO TFTs.

The electrical transport characteristics of typical *a-*IGZO TFTs prepared at the maximum process temperature of 150 °C are shown in Fig. 3. Figure 3b summarizes the major TFT performance parameters. First, the DRY sample did not exhibit a satisfactory performance, showing a field-effect mobility (μ_FE_) of ~4.2 cm^2^ V^−1^ s^−1^, threshold voltage (V_th_) of approximately −0.5 V, subthreshold swing (SS) of 0.45 V dec^−1^, and I_on_/I_off_ ≈ 10^7^, although hard saturation was observed. Here, V_th_ was defined as the gate voltage (V_G_) that induces a drain current (I_D_) of 1 nA obtained from 10 nA × L/W, while the μ_FE_ and SS values were estimated as follows:


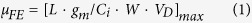



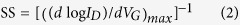


The L, W, g_m_, C_i_, and V_D_ are the channel length, channel width, transconductance, gate insulator capacitance per unit area, and drain voltage at the linear region, respectively^30^. In particular, this sample showed a high I-V hysteresis value (∆V_th,Hys_) of 8.85 V, likely due to the increased trap density arising from the inadequate atomic rearrangement and loose M–O bonding at low temperature. To obtain adequate conductivity of IGZO thin films, a high thermal annealing temperature (≥300 °C) is required for atomic rearrangement and strong M–O bonding. On the contrary, the low temperature wet-pulse annealed IGZO TFTs exhibited relatively low hysteresis curves together with relatively negative V_th_ values. This indicates that wet pulse annealing can effectively reduce the charge trap density of the oxide TFTs at a relatively low temperature, resulting in more conductive channel layers. The decrease in charge trap site density is a factor for increasing the carrier density due to electrons released from charge trap sites. V_th_ shifted positively from −12.5 V (20WET) to −3.7 V (286WET) with increasing wet pulse times. In addition, the μ_FE_ and the I_on_/I_off_ ratio were significantly improved. The 86WET sample exhibited an excellent TFT performance with μ_FE_ ≈ 13.3 cm^2^ V^−1^ s^−1^ and I_on_/I_off_ ratio ≈ 10^8^, as well as a reduced I-V hysteresis. This result could be ascribed to the reduced fractions of hydroxide and impurities, and to the strong M–O bonding^29^,^31^, which caused increased electrical conductivity. However, the high pulse number used for the ≥286WET sample resulted in a negative shift of V_th_ and a degraded SS value, implying that an optimum supply of H_2_O is necessary for low-temperature wet pulse annealing. Indeed, because the increased SS value is related to the increased number of the gate dielectric/IGZO semiconductor interfacial traps or H_2_O related impurities, the use of H_2_O in the annealing process of the 430WET sample may have affected the chemical bonding up to the gate dielectric interface.

**Figure 4:**
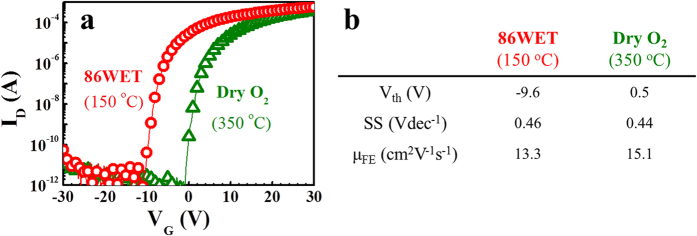
(**a**) Transfer curves for the 86WET (injection number: 86; annealing temperature: 150 °C) and dry-annealed (350 °C) thin film transistors (TFTs: at V_D_ = 10 V). (**b**) Summary of electrical parameters extracted from these TFTs.

The 86WET sample exhibited a quite good TFT performance, despite the low process temperature; thus, we compared it with a typical *a-*IGZO TFT by performing thermal annealing at 350 °C and in dry ambient, as this is the general annealing temperature region providing *a-*IGZO TFTs with good performance. This reference sample exhibited μ_FE_ ≈ 15.1 cm^2^ V^−1^ s^−1^, V_th_ ≈ 0.5 V, and SS ≈ 0.44 V dec^−1^, confirming that our new process based on wet pulse annealing produced samples with comparable TFT performance, despite the thermal annealing conducted at 150 °C, as shown in Fig. 4. Based on these results, we believe that the concept behind the wet pulse annealing process opens up new possibilities for high-performance flexible *a-*IGZO TFTs, as the vacuum deposition process provides great advantages such as the possibility of large scale fabrication, highly uniform metal cation distribution, and robust channel formation, compared with other solution process recently developed.

**Figure 5:**
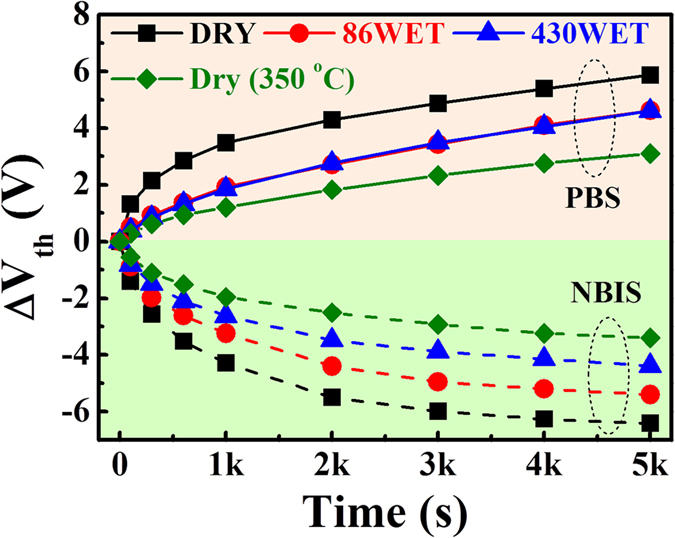
Summary of positive gate bias stress (PBS) and negative gate bias illumination stress (NBIS) tests as a function of time for DRY (dry annealed), 86WET (injection number: 86), and 430WET (injection number: 430) thin film transistors (TFTs) processed at 150 °C, along with a 350 °C dry processed TFT (reference). PBS and NBIS conditions were as follows: i) PBS: V_G_ = +10 V, duration time = 5000 s; ii) NBIS: V_G_ = −10 V, wavelength = 550 nm, power intensity = 0.1 mW cm^−2^, duration time = 5000 s.

To investigate the bias stability of *a-*IGZO TFTs, positive bias stress (PBS) and negative gate bias illumination stress (NBIS) tests were performed at room temperature (Fig. 5). Although the samples processed with wet pulse annealing at low temperature showed relatively lower stability than the samples annealed at 350 °C, a PBS and NBIS performance improvement was observed for the wet pulse annealed *a-*IGZO TFTs, compared with the dry-annealed sample. The 86WET sample exhibited a reduction in V_th_ by approximately −5.4 V upon application of light-illuminated NBS for 5000 s, while the sample 430WET exhibited a V_th_ shift of −4.4 V. In the case of the PBS, however, the dependence on the wet pulse conditions was negligible. In addition, the active defect creation at the gate dielectric/*a-*IGZO interface could be ignored, as the SS values did not change significantly during the PBS and NBIS tests, as shown in Supplementary (not shown here)^31^.

**Figure 6:**
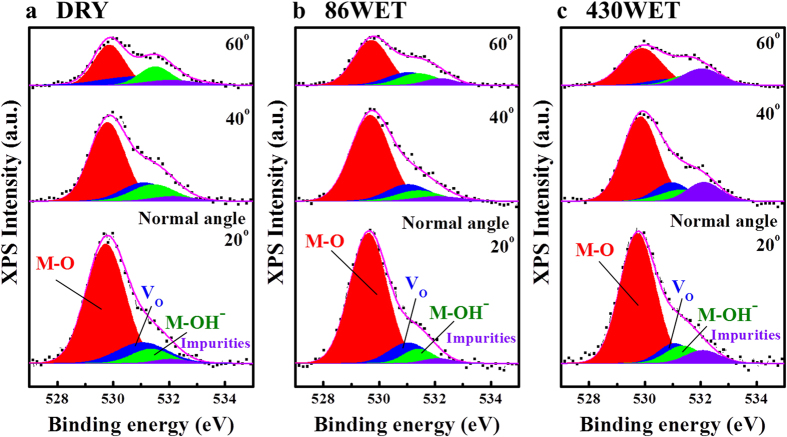
O 1s angle-resolved X-ray photoelectron spectra and fitting curves for (**a**) DRY (dry annealed), (**b**) 86WET (injection number: 86), and (**c**) 430WET (injection number: 430) amorphous IGZO films. Here, the O 1s spectra were deconvoluted into four fitting curves with Gaussian and Lorentzian functions.

**Figure 7:**
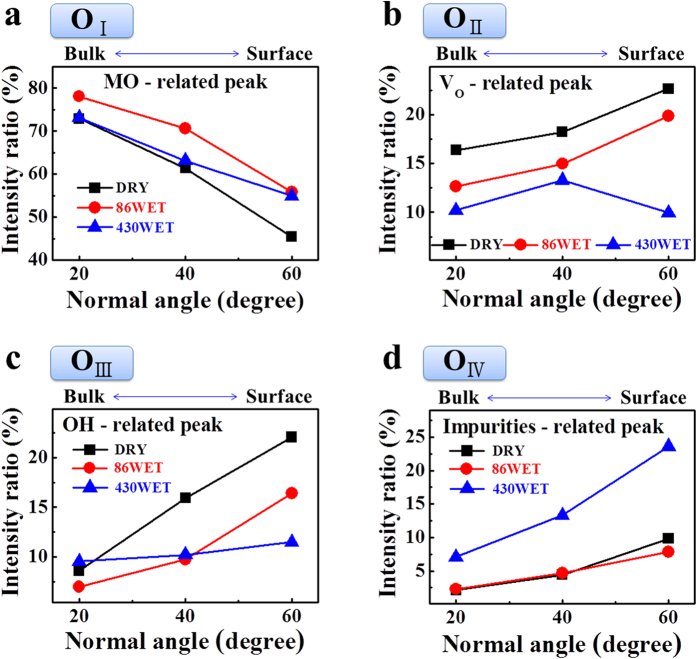
Contribution ratio obtained from the integrated intensities of angle-resolved X-ray photoelectron spectroscopy fitting curves related to (**a**) O_I_ (metal-oxygen), (**b**) O_II_ (oxygen deficiency), (**c**) O_III_ (hydroxide), and (**d**) O_IV_ (impurities or residues) depending on the normal angle.

To investigate in detail the oxygen-related bonding behavior, we additionally performed angle-resolved X-ray photoelectron spectroscopy (AR-XPS) measurements, as this technique allows characterizing the surface chemical states as functions of the depth of the ultrathin layers^32^. The XPS signal was collected in the approximate range of 20–60° from the normal of the sample surface; a high normal angle indicates near surface chemical information. The C–C component was set to a reference binding energy of 284.5 eV for the C 1s spectrum^33^. The O 1s peaks were deconvoluted into four fitting curves with Gaussian and Lorentzian functions, as shown in Figs 6 and 7. The four curves corresponded to the oxygen in the metal-oxide lattice (M–O; 529.7 eV, O_I_), oxygen deficient bonding (V_O_; 531.1 eV, O_II_), oxygen in the hydroxide (M–OH^−^; 531.4 eV, O_III_), and residues or impurities (532.1 eV, O_IV_)^34^,^35^; their intensity ratios are summarized in Fig. 7. By increasing the normal angle (close to the surface), the M–O related O_I_ contribution continuously decreased, while the ratio of O_III_ and O_IV_ were enhanced in all the samples, indicating that the hydroxide-related oxygen bonding and unwanted impurities were mainly distributed on the surface. The O_IV_ signal showed the strongest intensity in the surface region of the 430WET sample, owing to weakly bonded oxygen and H_2_O-related impurities^31^ due to an excess in the supply of H_2_O^36^. Neverthless, the high wet pulse number in the 430WET sample suppressed the generation of oxygen vacancies on the surface. On the other hand, the O_II_, O_III_, and O_IV_ in the DRY and 86WET samples showed identical trends depending on the normal angle. Notably, compared with the DRY sample, the results of the O_II_ and O_III_ peaks revealed a reduction in oxygen vacancies and hydroxide-related bonding^35^, due to the use of wet pulse annealing. The hydroxide content in *a-*IGZO films is an important factor that affects the electrical conductivity by trapping electron charges; thus, the considerable TFT performance improvement of the 86WET sample was ascribed to the suppression of hydroxide formation^27^,^28^. Furthermore, the 86WET sample showed the highest contribution in terms of strong M–O bonding (Fig. 7a). As shown in Fig. 5, our samples produced by wet pulse annealing showed an enhancement in the NBIS test, compared with the DRY sample. In general, these results were attributed to the photoionization and subsequent transition of electronic states of the oxygen vacancies. Therefore, the improvement in the NBIS test occurred in accordance with the suppression of the oxygen vacancies; the O_II_ peak area followed the order of 430WET < 86WET < DRY, and the 430WET sample with excess wet pulse exhibited a smaller V_th_ shift than 86WET.

**Figure 8:**
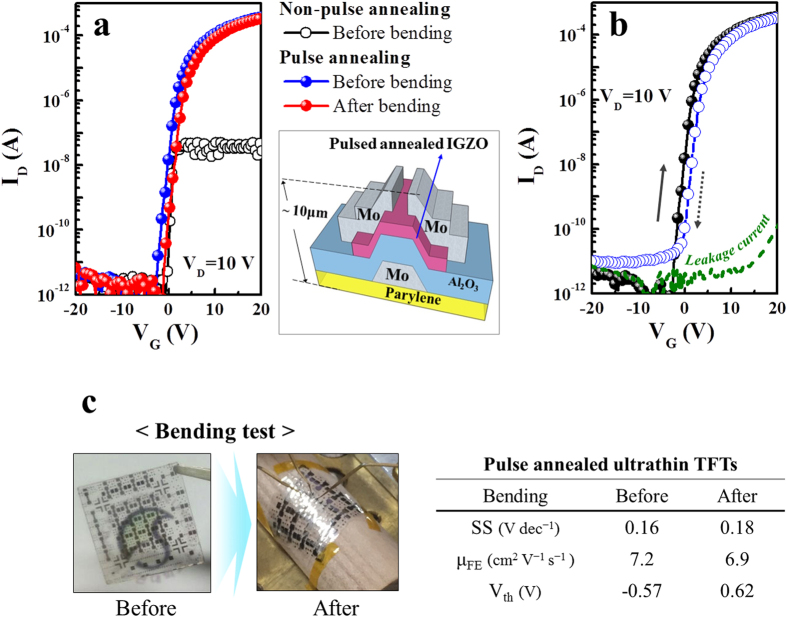
(**a**) Transfer curves of ultrathin flexible amorphous IGZO thin film transistors fabricated with and without wet pulsed annealing (at V_D_ = 10 V); (**b**) hysteresis characteristics; (**c**) bending performance.

Consequently, the low-temperature wet pulse annealing provided excellent *a-*IGZO TFTs comparable with those annealed at temperatures higher than 300 °C. Finally, we fabricated an ultrathin *a-*IGZO TFT by using a similar process on a plastic substrate, where the 86WET annealing process was applied after the definition of *a-*IGZO channel. The total thickness of the device including the substrate was around ~10 μm, and water-soluble polyvinyl alcohol (PVA) was used as a sacrificial layer to detach the thin Parylene substrate with *a-*IGZO TFT and the rigid glass holder, while the maximum process temperature was 150 °C. More details on this process, which we named “floating process”, will be discussed in another paper. As shown in Fig. 8, the ultrathin flexible *a-*IGZO TFT with the bottom-gate configuration exhibited good TFT performance with μ_FE_ ≈ 7.2 cm^2^ V^−1^ s^−1^, V_th_ ≈ −0.57 V, and SS ≈ 0.16 V dec^−1^. In addition, this TFT showed relatively low hysteresis (ΔV_th_ ~ 1.9 V) behavior. Moreover, the *a-*IGZO TFT had almost identical transfer curves before and after the bending test with a bending radius of 10 mm, while no sample obtained by wet pulse annealing showed poor conductivity or inadequate electrical performance.

## Discussion

We proposed a periodically pulsed wet annealing technique for low-temperature (150 °C) annealing of *a-*IGZO TFTs showing relatively good electrical performance/stability and comparable to TFTs obtained by conventional dry annealing at 350 °C; in this method, the supply amount of H_2_O could be artificially controlled by the pulse number during thermal annealing. The electrical characteristics of *a-*IGZO TFTs fabricated with 86 cycles at the maximum process temperature of 150 °C exhibited μ_FE_ ≈ 13.3 cm^2^ V^−1^ s^−1^ and an I_on_/I_off_ ratio ≈ 10^8^, as well as enhanced PBS and NBIS stability. From the AR-XPS chemical analysis, we found that appropriate H_2_O pulses suppressed the formation of oxygen-related defects and hydroxide acting as electron trap sites, providing strong M–O bonding. Consequently, an ultrathin flexible *a-*IGZO TFT on a plastic substrate was successfully fabricated and exhibited good electrical and bending performances.

## Methods

### Fabrication of InGaZnO TFTs

Staggered bottom-gate type *a-*IGZO TFTs were fabricated on heavily doped *p-*type Si substrates with thermally oxidized SiO_2_ (200 nm). The channel length (L) and width (W) were 50 and 500 μm, respectively. The *a-*IGZO channel-layers (~60 nm) were grown at room temperature by radio frequency (rf) magnetron sputtering by using a high purity 4-inch InGaZnO target (In:Ga:Zn = 2:1:2) at the O_2_/Ar gas ratio of 0.07 and rf power of 150 W. The *a-*IGZO channel-layers were defined by conventional photolithography and wet etching processes. Furthermore, 100-nm-thick molybdenum (Mo) layers were deposited by direct current magnetron sputtering to be used as source and drain electrodes.

Prior to the Mo deposition, low-temperature thermal annealing was performed at 150 °C with and without wet pulses. The annealing temperature and time for the TFT fabrication were set to 150 °C and 150 min, respectively. Our pulse annealing consisted of periodic injections of H_2_O and relatively long dry purges. As shown in Fig. 2, our pulse annealing used quick injection of water vapor for 0.1 s and dry treatment with different purge times in one cycle. The three different samples were selected; 20WET (injection number: 20), 86WET (injection number: 86), 286WET (injection number: 286), 430WET (injection number: 430) and DRY (injection number: 0; typically dry annealing).

To demonstrate the effectiveness of the wet pulse annealing at 150 °C, we produced staggered bottom-gate and top-gate type ultrathin *a-*IGZO TFTs with pulsed wet annealing on Parylene substrates. Here, the formation of ultrathin Parylene substrates with a thickness of 10 μm was conducted by adopting a floating process using a sacrificial water soluble PVA; a more detailed procedure will be described elsewhere. Here, 80-nm-thick Al_2_O_3_ dielectric layers were deposited by atomic layer deposition at 150 °C, while 100-nm-thick Mo layers were used as the gate and source/drain electrodes; in addition, 60-nm-thick *a-*IGZO films were used as the channel-layer and were annealed under the optimized conditions used for the 86WET sample.

### Characterization of Thin Films and TFTs

The thicknesses of the channel-layers and metal electrodes were measured by an alpha-step surface profiler (XP-100, Ambios Technology, Inc.). The chemical bonding of the *a-*IGZO films was characterized by AR-XPS (Theta Probe, Thermo Fisher Scientific Co.). The TFT performances and stability tests, including PBS and NBIS, were performed with a semiconductor parameter analyzer (HP 4145B). For the light source to apply the illumination stress, a 150 W Xe arc lamp (LS-150, ABET Technologies Inc.) and a monochromator (Monora 200, DONGWOO OPTRON Co., Ltd.) were used. The optical power of the monochromatic light was measured using a UV-enhanced Si photodetector and was controlled to be 0.1 mW cm^−2^.

## Additional Information

**How to cite this article**: Kim, Y. K. *et al.* Periodically pulsed wet annealing approach for low-temperature processable amorphous InGaZnO thin film transistors with high electrical performance and ultrathin thickness. *Sci. Rep.* **6**, 26287; doi: 10.1038/srep26287 (2016).

## Supplementary Material

Supplementary Information

## References

[b1] Mo Y. G. *et al.* Amorphous-oxide TFT backplane for large-sized AMOLED TVs. J. Soc. Inf. Disp. 19, 16–20 (2011).

[b2] Fortunato E., Barquinha P. & Martinsv R. Oxide Semiconductor Thin-Film Transistors: A Review of Recent Advances. Adv. Mater. 24, 2945–2986 (2014). 22573414 10.1002/adma.201103228

[b3] Park J. S. *et al.* Flexible full color organic light-emitting diode display on polyimide plastic substrate driven by amorphous indium gallium zinc oxide thin-film transistors. Appl. Phys. Lett. 95, 013503 (2009).

[b4] Smith J. T., Shah S. S., Goryll M., Stowell J. R. & Allee D. R. Flexible ISFET Biosensor Using IGZO Metal Oxide TFTs and an ITO Sensing Layer. IEEE Sens. J. 14, 937–938 (2014).

[b5] Kumomi H., Kamiya T. & Hosono H. Advances in Oxide Thin-Film Transistors in Recent Decade and Their Future. ECS Trans. 67, 3–8 (2015).

[b6] Lee G. J. *et al.* High performance, transparent a-IGZO TFTs on a flexible thin glass substrate. Semicond. Sci. Technol. 29, 035003 (2014).

[b7] Kwon J. Y. *et al.* Bottom-Gate Gallium Indium Zinc Oxide Thin-Film Transistor Array for High-Resolution AMOLED Display. IEEE Electron Device Lett. 29, 1309 (2008).

[b8] Xu H. *et al.* A flexible AMOLED display on the PEN substrate driven by oxide thin-film transistors using anodized aluminium oxide as dielectric. J. Mater. Chem. C 2, 1255–1259 (2014).

[b9] Kim J. S. *et al.* Effects of low-temperature (120 °C) annealing on the carrier concentration and trap density in amorphous indium gallium zinc oxide thin film transistors. J. Appl. Phys. 116, 245302 (2014).

[b10] Fuh C. S., Liu P. T., Huang W. H. & Sze S. M. Effect of Annealing on Defect Elimination for High Mobility Amorphous Indium-Zinc-Tin-Oxide Thin-Film Transistor. IEEE Electron Device Lett. 35, 1103–1105 (2014).

[b11] Li L. *et al.* Effect of thermal annealing on the properties of transparent conductive In–Ga–Zn oxide thin films. J. Vac. Sci. Technol. A 32, 021506 (2014).

[b12] Kimura M., Nakanishi T., Nomura K., Kamiya T. & Hosono H. Trap densities in amorphous- In Ga Zn O 4 thin-film transistors. Appl. Phys. Lett. 92, 133512 (2008).

[b13] Furuta M. *et al.* Analysis of Hump Characteristics in Thin-Film Transistors With ZnO Channels Deposited by Sputtering at Various Oxygen Partial Pressures. IEEE Electron Device Lett. 31, 1257–1259 (2010).

[b14] Cho S. W., Yun M. G., Ahn C. H., Kim S. H. & Cho H. K. Bi-layer Channel Structure-Based Oxide Thin-Film Transistors Consisting of ZnO and Al-Doped ZnO with Different Al Compositions and Stacking Sequences. Electron. Mater. Lett. 11, 198–205 (2015).

[b15] Park S. Y. *et al.* The Effect of Annealing Ambient on the Characteristics of an Indium–Gallium–Zinc Oxide Thin Film Transistor. J. Nanosci. Nanotechnol. 11, 6029–6033 (2011). 22121652 10.1166/jnn.2011.4360

[b16] Fuh C. S. *et al.* Effects of Microwave Annealing on Nitrogenated Amorphous In-Ga-Zn-O Thin-Film Transistor for Low Thermal Budget Process Application. IEEE Electron Device Lett. 34, 1157–1159 (2013).

[b17] Kim Y. H. *et al.* Flexible metal-oxide devices made by roomtemperature photochemical activation of sol–gel films. Nature 489, 128–132 (2012). 22955624 10.1038/nature11434

[b18] Ji K. H. *et al.* Effect of high-pressure oxygen annealing on negative bias illumination stressinduced instability of InGaZnO thin film transistors. Appl. Phys. Lett. 98, 103509 (2011).

[b19] Nomura K., Kamiya T., Ohta H., Hirano M. & Hosono H. Defect passivation and homogenization of amorphous oxide thin-film transistor by wet O 2 annealing. Appl. Phys. Lett. 93, 192107 (2008).

[b20] Ide K., Kikuchi Y., Nomura K., Kamiya T. & Hosono H. Effects of low-temperature ozone annealing on operation characteristics of amorphous In–Ga–Zn–O thin-film transistors. Thin Solid Films 520, 3787–3790 (2012).

[b21] Rim Y. S., Jeong W. H., Ahn B. D. & Kim H. J. Defect reduction in photon-accelerated negative bias instability of InGaZnO thin-film transistors by high-pressure water vapor annealing. Appl. Phys. Lett. 102, 143503 (2013).

[b22] Nomura K., Kamiya T., Hirano M. & Hosono H. Origins of threshold voltage shifts in room-temperature deposited and annealed a – In – Ga – Zn – O thin-film transistors. Appl. Phys. Lett. 95, 013502 (2009).

[b23] Kikuchi Y. *et al.* Device characteristics improvement of a-In–Ga–Zn–O TFTs by low-temperature annealing. Thin Solid Films 518, 3017–3021 (2010).

[b24] Fakhri M., Johann H., Görrn P. & Riedl T. Water as Origin of Hysteresis in Zinc Tin Oxide Thin-Film Transistors. ACS Appl. Mater. Interfaces 4, 4453–4456 (2012). 22939293 10.1021/am301308y

[b25] Lee K. H. *et al.* The effect of moisture on the photon-enhanced negative bias thermal instability in Ga–In–Zn–O thin film transistors. Appl. Phys. Lett. 95, 232106 (2009).

[b26] Watanabe K. *et al.* Surface reactivity and oxygen migration in amorphous indium-gallium-zinc oxide films annealed in humid atmosphere. Appl. Phys. Lett. 103, 201904 (2013).

[b27] Jeong S. H., Kim D. J., Lee S., Park B. K. & Moona J. H. Organic-inorganic hybrid dielectrics with low leakage current for organic thin-film transistors. Appl. Phys. Lett. 89, 092101 (2006).

[b28] Chua L. L. *et al.* General observation of n-type field-effect behaviour in organic semiconductors. Nature 434, 194–199 (2005). 15758994 10.1038/nature03376

[b29] Kim M. G., Kanatzidis M. G., Facchetti A. & Marks T. J. Low-temperature fabrication of high-performance metal oxide thin-film electronics via combustion processing. Nat. Mater. 10, 382–388 (2011). 21499311 10.1038/nmat3011

[b30] Yun M. G. *et al.* Dual Electrical Behavior of Multivalent Metal Cation-Based Oxide and Its Application to Thin-Film Transistors with High Mobility and Excellent Photobias Stability. ACS Appl. Mater. Interfaces 7, 6118–6124 (2015). 25714371 10.1021/am5085836

[b31] Jeong J. K., Yang H. W., Jeong J. H., Mo Y. G. & Kim H. D. Origin of threshold voltage instability in indium-gallium-zinc oxide thin film transistors. Appl. Phys. Lett. 93, 123508 (2008).

[b32] Ueoka Y. *et al.* Analysis of electronic structure of amorphous InGaZnO/SiO2 interface by angleresolved X-ray photoelectron spectroscopy. J. Appl. Phys. 114, 163713 (2013).

[b33] Moulder J. F. Handbook of X-ray Photoelectron Spectroscopy (ed. Chastain J.) 216-217, 230-232 (Physical Electronics, Inc., 1995).

[b34] Yu X. *et al.* Ultra-Flexible, “Invisible” Thin-Film Transistors Enabled by Amorphous Metal Oxide/Polymer Channel Layer Blends. Adv. Mater. 27, 2390–2399 (2015). 25712894 10.1002/adma.201405400

[b35] Jeong S. H. *et al.* Metal salt-derived In–Ga–Zn–O semiconductors incorporating formamide as a novel co-solvent for producing solution-processed, electrohydrodynamic-jet printed, high performance oxide transistors. J. Mater. Chem. C 1, 4236–4243 (2013).

[b36] Park J. S., Jeong J. K., Chung H. J., Mo Y. G. & Kim H. D. Electronic transport properties of amorphous indium-gallium-zinc oxide semiconductor upon exposure to water. Appl. Phys. Lett. 92, 072104 (2008).

