# Supplementary material for: Periodically pulsed wet annealing approach for low-temperature processable amorphous InGaZnO thin film transistors with high electrical performance and ultrathin thickness
Source: Sci Rep. 2016 May 20;6:26287. doi: 10.1038/srep26287 (PMC4873798; doi:10.1038/srep26287)
Supplement: Supplementary Information [file srep26287-s1.pdf]

Supplementary Information

Scientific reports

Periodically pulsed wet annealing approach for low-temperature processable amorphous InGaZnO thin film transistors with high electrical performance and ultrathin thickness

Ye Kyun Kim, Cheol Hyoun Ahn*, Myeong Gu Yun, Sung Woon Cho, Won Jun Kang, and Hyun Koun Cho*

School of Advanced Materials Science and Engineering, Sungkyunkwan University, 2066 Seobu-ro, Jangan-gu, Suwon, Gyeonggi-do, 16419, Republic of Korea

**Corresponding Atihors: e-mail (c2hyoun2@skku.edu, chohk@skku.edu)*

Table S1. State-of-the-art IGZO TFTs that are wet-base0annealed. Other annealing methods are also summarized.

Reference	Channel layer	Annealing			TFT Performance			Note
		Method	Ambient	Temperature [°C]	V_{th} [V]	μ [$cm^2\ V^{-1}\ s^{-1}$]	SS [$V\ dec^{-1}$]	
Domen, K. et al.[2]	IGZO, Sputtering	Wet	Continuous wet vapor	200	1.8	11.3 (sat)	0.2	▶ High temperature<br>▶ No demonstration of flexible TFTs
Shin, H. S. et al.[3]	IGZO, Sputtering	Wet	Continuous wet vapor	300	2.4	7.7 (FE)	0.21	▶ High temperature<br>▶ No demonstration of flexible TFTs
		Wet	Continuous wet vapor	300	0.3	13.2 (FE)	0.24	▶ High temperature<br>▶ No demonstration of flexible TFTs
Ahn, B. D. et al.[4]	IGZO, Sputtering	Wet	Continuous wet vapor	300	3	11.4 (FE)	0.76	▶ High temperature<br>▶ No demonstration of flexible TFTs
Yasuno, S. et al.[5]	IGZO, Sputtering	Wet	Continuous wet vapor	350	-0.3	16.5 (FE)	0.20	▶ High temperature<br>▶ No demonstration of flexible TFTs
Ji, K. H. et al.[6]	IGZO, Sputtering	High pressure O_2	O_2 of 0.5-10 atm	250	0.8-2.0	19.1-22.3 (FE)	0.25-0.32	▶ High temperature<br>▶ No demonstration of flexible TFTs
Fuh, C. -S. et al.[7]	IGZO, Sputtering	Microwave	Not mentioned	Not mentioned	2.9	4.2	0.4	▶ No demonstration of flexible TFTs
Kim, Y. -H. et al.[8]	IGZO, Sol-gel	Deep UV irradiation	N_2	<150	1.9	8.8 (sat)	0.17	▶ Only applicable to sol-gel method
This Research	IGZO, Sputtering	**Wet pulse**	**Pulsed wet vapor**	**150**	-0.6	7.2 (FE)[9]	0.19	▶ Novel low-temperature process<br>▶ Flexible and ultrathin TFTs

1. Domen, K. *et al.* Positive gate bias instability induced by diffusion of neutral hydrogen in amorphous In–Ga–Zn–O thin-film transistor. *IEEE Electron Device Lett.* **35**, 832-834 (2014)
2. Shin, H. S. *et al.* Effects of high-pressure H_2O annealing on amorphous IGZO thin-film transistors. *Phys. Status Solidi A.* **208**, 2231-2234 (2011)
3. Ahn, B. D. *et al.* Thin-film transistor behaviour and the associated physical origin of water-annealed In–Ga–Zn oxide semiconductor. *J. Phys. D: Appl. Phys.* **45**, 415307 (2012)
4. Yasuno, S. *et al.* Correlation of photoconductivity response of amorphous In–Ga–Zn–O films with transistor performance using microwave photoconductivity decay method. *Appl. Phys. Lett.* **98**, 102107 (2011)
5. Ji, K. H. *et al.* Effect of high-pressure oxygen annealing on negative bias illumination stress induced instability of IGZO thin film transistors. *Appl. Phys. Lett.* **98**, 103509 (2011)
6. Fuh C. -S. et al. Effects of Microwave Annealing on Nitrogenated Amorphous In-Ga-Zn-O Thin-Film Transistor for Low Thermal Budget Process Application. *IEEE Electron Device Lett.* **34**, 1157-1159 (2013).
7. Kim Y. -H. et al. Flexible metal-oxide devices made by room temperature photochemical activation of sol–gel films. *Nature* **489**, 128-132 (2012).

8. (FE) field-effect mobility
9. (sat) saturation mobility

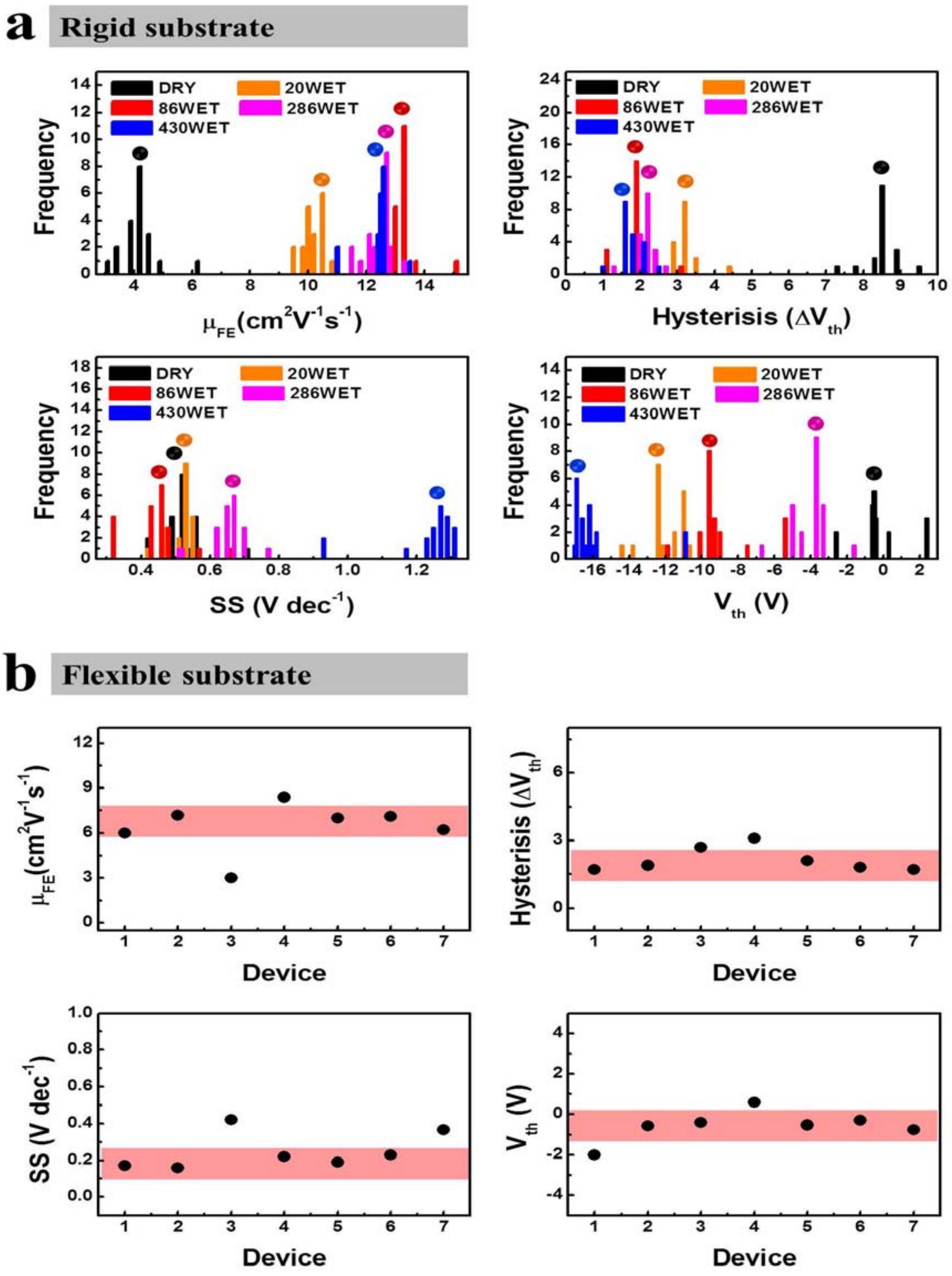

Figure S1. a) Statistical data for IGZO TFT devices fabricated with different annealing conditions on rigid substrates. b) Figures of merit for ultra-thin flexible IGZO TFTs.

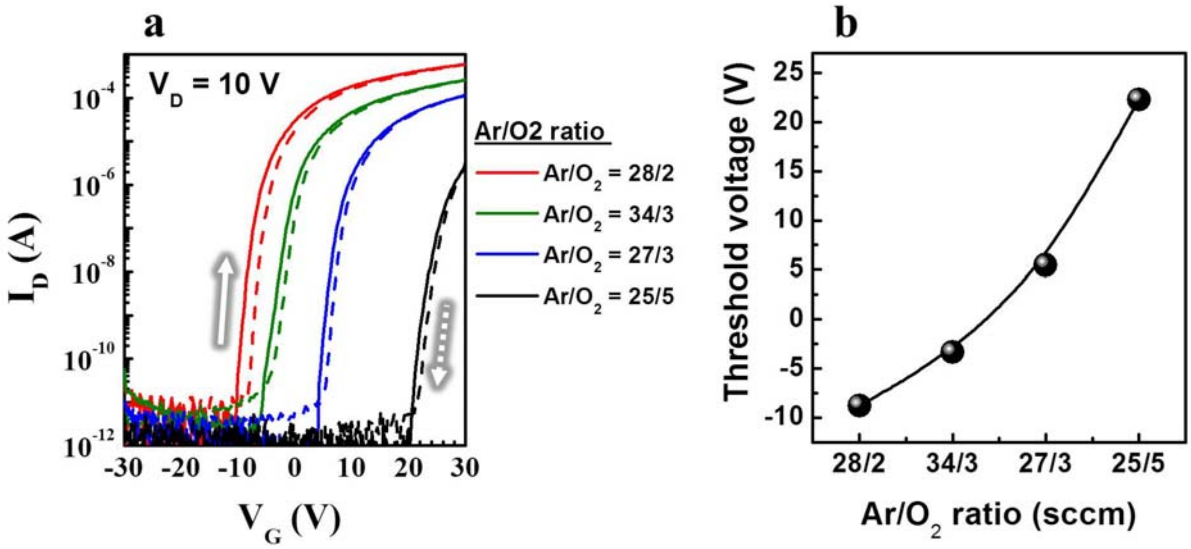

Figure S2. Electrical performances of 86WET IGZO TFTs prepared at different oxygen partial pressures during channel deposition; (a) transfer curves and hysteresis characteristics, and (b) variation of threshold voltage.

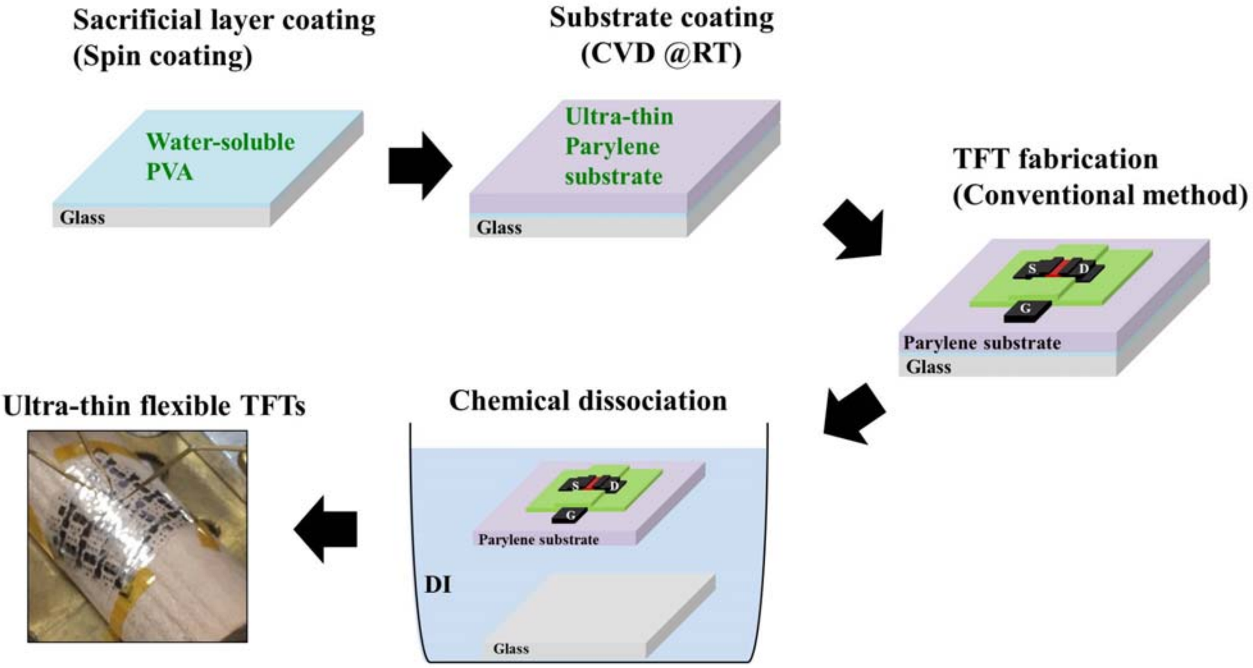

Figure S3. Process flow for the fabrication of ultra-thin flexible TFTs with ~10 μm thickness.
